# Does minimally invasive pylorus-preserving pancreaticoduodenectomy (MI-PPPD) triumph over open pylorus-preserving pancreaticoduodenectomy (O-PPPD)? A systematic review and meta-analysis

**DOI:** 10.1097/JS9.0000000000004734

**Published:** 2026-01-12

**Authors:** Benedict D.C. Ong, Wesford J.W.H. Goh, Marie M.T. Tan, Sean Loke, Jenelle R.Y. Kan, Jacob K.W. Yeo, Jia-Hao Law, Alfred W.C. Kow

**Affiliations:** a Yong Loo Lin School of Medicine, National University of Singapore, Singapore; b Division of Hepatobiliary and Pancreatic Surgery and Liver Transplantation, University Surgical Cluster, National University Hospital, Singapore

**Keywords:** laparoscopic, minimally invasive surgery, open, pylorus-preserving pancreatoduodenectomy, robotic, Whipple

## Abstract

**Background:**

PPPD is purported to be better than the Whipple procedure as it preserves full gastric capacity. Approaching this surgery using the MIS technique [minimally invasive pylorus-preserving pancreaticoduodenectomy (MI-PPPD)] may confer additional advantages compared to the open method. However, literature comparing MI-PPPD to open pylorus-preserving pancreaticoduodenectomy (O-PPPD) is scarce. This study seeks to review the efficacy and long-term outcomes of MI-PPPD against O-PPPD.

**Materials and Methods:**

This PRISMA-adherent review involved a systematic search of PubMed, Scopus, Cochrane, and Embase for studies evaluating the outcomes of MI-PPPD compared to O-PPPD. Two independent reviewers extracted the summary data from included studies. Random effects meta-analyses were used for analysis. All analyses were performed in RStudio (Posit Software, PBC, Boston, Massachusetts) Version 4.3.2. Artificial intelligence was not used for research and manuscript development.

**Results:**

Nine articles with 682 patients (391 in the MI-PPPD group and 291 in the O-PPPD group) were included. Postoperatively, there was no statistically significant differences in postoperative complications [risk ratio (RR) = 1.00, 95% CI: 0.69; 1.46], length of hospital stay [mean difference (MD) = −0.21, 95% CI: −0.53; 0.11], postoperative pancreatic fistula formation (RR = 0.78, 95% CI: 0.59; 1.05), delayed gastric emptying (RR = 0.63, 95% CI: 0.35; 1.14), postpancreatectomy hemorrhage (RR = 1.38, 95% CI: 0.77; 2.46), and 90-day mortality (RR = 2.42, 95% CI: 0.92; 6.34). Perioperatively, MI-PPPD had nonsignificant reduction in estimated blood loss (MD = −129.07, 95% CI: −579.35; −312.20) and nonsignificant difference in operative time (MD = 0.38 min, 95% CI: −0.18; 0.95). Oncological outcomes revealed equivalent outcomes in *R*_0_ resection (MD = 0.50%, 95% CI: 0.07; 3.82) and lymph node yield (MD = −1.31, 95% CI: −2.83; 0.22).

**Conclusion:**

MI-PPPD is noninferior to O-PPPD. Whilst MI-PPPD outcomes have been influenced by its high complexity and steep learning curve, this approach is believed to be on an upwards trajectory with the potential to surpass O-PPPD. Investment toward MI-PPPD training is recommended given the potential benefits it brings to patients.

## Introduction

Pylorus-preserving pancreatoduodenectomy (PPPD) is a modification of the pancreaticoduodenectomy (PD), typically known as the Whipple procedure[1]. Some purported key benefits of pylorus preservation in PPPD include reduced risk of delayed gastric emptying (DGE)[2], dumping syndrome, bile reflux gastritis, and postoperative gastrointestinal dysfunction^[3,4]^. Moreover, preservation of the gastric antrum may improve weight and nutritional maintenance over time[5]. However, the multitude of benefits aimed at improving the patient’s quality of life is accompanied by significant technical difficulties. With improvements in surgical techniques, PPPD has been progressively adopted, with literature demonstrating improvement in perioperative outcomes, postoperative outcomes, and comparable oncological outcomes^[6,7]^. This warrants an isolated assessment of PPPD and its approaches, if preserving these intended advantages is indeed possible.

Given the popularity of minimally invasive surgery (MIS), laparoscopic and robotic-assisted techniques have unsurprisingly found their way into pancreatic surgery. Key advantages of the MIS approach include small incisions that translate to lower risks of wound infection[8], decreased postoperative pain, and consequently faster postoperative recovery[8]. Some reported improved “quality of life” outcomes include better cosmesis and potentially earlier recovery with shorter duration of hospitalization[9]. However, MIS is technically complex and involves longer operative times and a much steeper learning curve[10]. While some studies report favorable surgical outcomes and reduced hospital stays[11], there is currently limited level 1 evidence to support its widespread adoption (including the prematurely terminated LEOPARD-2 trial)[12].

The conventional open approach, characterized by large abdominal incisions to create space for access and complex surgical maneuver offers direct visualization of the surgical site of interest, unrestricted tissue manipulation with enhanced speed, range of motion, and dexterity, and direct tissue palpation with tangible tactile feedback^[8,13]^. Some claim that this is a more suitable technical approach for PPPD in view of its complexity.

Theoretical benefits of MIS very much align with the goals of PPPD – ultimately seeking to improve the patient’s quality of life. However, the meaningful advantage of generic MIS approaches has yet to be certainly transferred over to pancreatic head surgeries, particularly PPPD. Current evidence for minimally invasive pylorus-preserving pancreaticoduodenectomy (MI-PPPD) remains scarce and conflicting – there has not been a study that directly and extensively compares both approaches in this procedure. As a rigorous, evidence-based comparison between MI-PPPD and open pylorus-preserving pancreaticoduodenectomy (O-PPPD) is lacking, this systematic review and meta-analysis aims to evaluate the perioperative, postoperative, and oncological outcomes between MI-PPPD and O-PPPD. Crucial insights on the efficacy and safety of MI-PPPD inform the role of MIS in PPPD, guide surgical decision-making, and improve patient care. This meta-analysis also assesses if the MIS approach can achieve and maintain additional benefits derived from PPPD compared to PD given the technical complexity.

## Materials and methods

### Protocol and guidance

The Preferred Reporting Items for Systematic Reviews and Meta-Analyses (PRISMA) Checklist and A Measurement Tool to Assess Systematic Reviews 2 (AMSTAR 2) guidelines were utilised to guide the reporting of this systematic review, as included in Supplemental Digital Content Tables S1 and S2, available at: http://links.lww.com/JS9/G593, respectively^[14,15]^. The protocol was registered on PROSPERO, and no deviations from the protocol were made[16]. The authors also emphasize that no artificial intelligence (AI) was used in the research and development of this manuscript per the TITAN Guidelines 2025 Checklist, as included in Supplemental Digital Content Table S3, available at: http://links.lww.com/JS9/G593^17^.

### Data sources and search strategy

A literature search was performed in established scientific databases, including PubMed, Embase, Cochrane, and Scopus. The search strategy combined search terms for PPPD and MIS. Database-controlled vocabulary was used to search for subject headings and synonyms with appropriate truncations for searching title, abstract, and author keywords. The search strategy was translated between each database. The full search strategy is reported in Supplemental Digital Content Table S4, available at: http://links.lww.com/JS9/G593. References from included studies and review papers were hand-searched. Case reports, case series, conference reports, and other grey literature were excluded. Only studies published after the year 2000 were included.

**HIGHLIGHTS**

- Minimally invasive pylorus-preserving pancreaticoduodenectomy (MI-PPPD) is a safe and effective approach when compared to open pylorus-preserving pancreaticoduodenectomy (O-PPPD) based on the existing literature.
- Postop complications and mortality are comparable between the two groups.
- Oncologic outcomes, *R*_0_ resection, and lymph node retrieval, are also comparable.
- MI-PPPD is forecasted to surpass O-PPPD with continued training and expertise.

### Study selection: inclusion and exclusion criteria

Two reviewers independently screened all articles for their eligibility according to the inclusion and exclusion criteria. Studies assessed as “relevant” but “unclear” in terms of eligibility were independently evaluated by a third reviewer. The full texts of all potentially eligible studies were then retrieved and reviewed by two independent reviewers, with any discrepancies resolved by a third reviewer. A total of nine studies were included, of which six were retrospective studies, and three were prospective studies.

Studies that assessed the intra-operative and postoperative outcomes of patients undergoing MI-PPPD or O-PPPD were evaluated. MI-PPPD includes both laparoscopic and robotic PPPD procedures. All interventional studies were included if they assessed (1) estimated blood loss, (2) operative time, (3) incidence of postoperative complications, (4) length of hospital stay, (5) incidence of postoperative pancreatic fistula, (6) incidence of DGE, (7) incidence of postpancreatectomy hemorrhage, (8) incidence of 90-day mortality, (9) rate of negative margin resections, (10) dissected lymph node yield, and (11) incidence of conversion from MI-PPPD to open surgery. Studies were excluded from the relevant analyses if they (1) solely consisted of patients undergoing O-PPPD, (2) solely published data on patients receiving other variations of PD, or (3) were reviews, case reports, case series, letters, comments, editorials, conference proceedings, trial registrations, and personal communications. To ensure a comprehensive review, both manual-searching and backward and forward reference searching of the included studies were also conducted.

### Data extraction and organization

Baseline patient information, including age, gender, and co-morbidities were collected. Disease information was collected as well. Where available, studies comparing the surgical outcomes of MI-PPPD against O-PPPD were pooled and compared in the double-arm analyses. The control group was defined as patients undergoing complete O-PPPD. Where comparative data were not available, studies solely reporting on the surgical outcomes of MI-PPPD were systematically reviewed to provide summative data.

Postsurgical complications were defined according to the Clavien–Dindo classification of surgical complications^18^. Relative risk of postoperative complications, postoperative pancreatic fistula formation, DGE, postpancreatectomy hemorrhage (PPH), and 90-day mortality were quantified by computing the risk ratio (RR). Negative margin resection rates were quantified by computing the odds ratio (OR). Estimated blood loss and dissected lymph node yield were quantified by computing the mean difference (MD). Operative time and length of hospital stay were quantified by computing the standardized mean difference (SMD).

### Data analysis

The *meta* and *metafor* packages were utilized for all analyses conducted on R (version 4.1.0). For dichotomous outcomes used, including postoperative complications, postoperative pancreatic fistula, DGE, postpancreatectomy hemorrhage, and 90-day mortality, meta-analyses were performed to compute the relative risk (measured using the RR compared to controls). For continuous outcomes, such as the operative time and length of hospital stay, the SMD was used as it provides a direct measure of the difference in operative time and length of hospital stay between both surgical approaches. For estimated blood loss and dissected lymph node yield, the MD was used. This analysis was conducted using the inverse variance method under a random-effects model to account for variability between studies. For both dichotomous and continuous outcomes, a random-effects model was applied due to the high clinical likelihood that the included study populations are heterogeneous. Between-study heterogeneity was evaluated using *τ*^2^ and *I*^2^ statistics, where an *I*^2^ value of 25% indicates low heterogeneity, 50% indicates moderate heterogeneity, and 75% indicates high heterogeneity. Two-sided *P*-values < 0.05 were regarded as indicating nominal statistical significance[17]. Publication bias was not assessed as there were fewer than ten studies used in the entire analysis[18].

### Risk of bias assessment

The methodological quality was evaluated using the AMSTAR 2 checklist, which includes the measurement of condition, appraisal of the inclusion criteria, reporting of the baseline characteristics, the reporting of outcomes, and the appropriateness of the statistical analysis[15]. The Newcastle–Ottawa Scale (NOS) was also used to evaluate methodological risk of bias at the study level[19]. Appraisals were performed by two reviewers independently, with any discrepancies resolved by the verdict of a third reviewer.

## Results

Nine studies comprising 682 patients were included in the final analysis, with 2^[20,21]^ being excluded when counting patient numbers^[20–28]^. 391 were in the MI-PPPD group, and 291 were in the O-PPPD group. The PRISMA flow diagram for the selection of studies was included in Supplemental Digital Content Figure S1, available at: http://links.lww.com/JS9/G593. Characteristics of the studies included were further described in Table 1. The final pathologic diagnoses in the MI-PPPD group are shown in Table 2. Comparison of the outcomes between MI-PPPD and O-PPPD were summarized and included in Table 3. The quality of the methodology was scored with the AMSTAR 2 tool (Supplemental Digital Content Table S2, available at: http://links.lww.com/JS9/G593). The quality of these nonrandomized studies was assessed with the NOS, and the level of bias of the studies were deemed “low” to “moderate” (Supplemental Digital Content Tables S5 and S6, available at: http://links.lww.com/JS9/G593). Three single-arm studies were not included in the formal pairwise meta-analyses (Supplemental Digital Content Figure S2, available at: http://links.lww.com/JS9/G593), and were instead systematically reviewed where appropriate.

**Table 1:** Study characteristics.

Study ID	Country	Study design	Sample size (MI-PPPD/O-PPPD)	Surgical indication, *n*	Single-center/multicenter	Surgeon’s experience level
Keck *et al* 2011^[24]^	Germany	Cohort	367 (9/358)	Pancreatic ductal adenocarcinoma (*n* = 2), ampullary carcinoma (*n* = 3), chronic pancreatitis (*n* = 1), cystic neoplasms of the pancreas (*n* = 1), others (*n* = 2)	Single-centric	Single surgeon with over 300 open pancreatic resections and extensive experience with reconstruction via pancreatogastrotomy
Wellner *et al* 2014^[22]^	Germany	Cohort	80 (40/40)	Pancreatic ductal adenocarcinoma (*n* = 14), periampullary adenocarcinoma (*n* = 14), cystic neoplasm of the pancreas (*n* = 24), neuroendocrine tumor (*n* = 8), chronic pancreatitis (*n* = 8), others (*n* = 12)	Multicentric	No record
Morelli *et al* 2023^[23]^	Italy	Cohort	60 (30/30)	Pancreatic ductal adenocarcinoma (*n* = 35), ampullary carcinoma (*n* = 7), cholangiocarcinoma (*n* = 3), intraductal papillary mucinous neoplasm (*n* = 5), pancreatic neuroendocrine tumor (*n* = 2), others (*n* = 8)	Single-centric	A single consulting academic surgeon working in a high-volume center for both robotic and pancreatic surgery
Wang *et al* 2015^[20]^	China	Cohort	10 (10/–)	No record	Single-centric	No record
Kim *et al* 2012^[25]^	South Korea	Cohort	100 (100/–)	Intraductal papillary mucinous neoplasm (*n* = 37), solid pseudopapillary neoplasm, (*n* = 17), pancreatic neuroendocrine tumor (*n* = 15), pancreatic ductal adenocarcinoma (*n* = 7), CNP (*n* = 7), ampullary carcinoma (*n* = 5), chronic pancreatitis (*n* = 1), cholangiocarcinoma (*n* = 1), others (*n* = 10)	Single-centric	Single senior and experienced surgeon
Cho *et al* 2009^[26]^	Japan	Case control	30 (15/15)	Pancreatic ductal adenocarcinoma (*n* = 3), cholangiocarcinoma (*n* = 4), ampullary carcinoma (*n* = 6), intraductal papillary mucinous neoplasm (*n* = 13), neuroendocrine tumor (*n* = 2), others^a^ (*n* = 2)	Single-centric	Laparoscopic pylorus-preserving pancreaticoduodenectomy was performed by one author who had experienced more than 30 laparoscopic hepatectomies, including hepatic lobectomies. Open pylorus-preserving pancreaticoduodenectomies were performed by three authors
Song *et al* 2015^[27]^	South Korea	Case control	186 (93/93)	Intraductal papillary mucinous neoplasm (*n* = 86), solid pseudopapillary neoplasm (*n* = 32), neuroendocrine tumor (*n* = 36), cystic neoplasms of the pancreas (*n* = 11), others (*n* = 21)	Single-centric	All six surgeons have performed more than 100 open pylorus-preserving pancreaticoduodenectomy procedures with relatively similar experience levels. Two surgeons performed laparoscopic pylorus-preserving pancreaticoduodenectomy, though most were performed by a single surgeon
Han *et al* 2019^[28]^	South Korea	Cohort	217 (104/113)	Pancreatic ductal adenocarcinoma (*n* = 69), neuroendocrine tumor (*n* = 8), intraductal papillary mucinous neoplasm, (*n* = 15), cholangiocarcinoma (*n* = 66), ampulla of Vater carcinoma (*n* = 36), others (*n* = 23)	Single-centric	No record
Cai *et al* 2021^[21]^	China	Cohort	144 (83/–)	No record	Single-centric	No record

MI-PPPD, minimally invasive pylorus-preserving pancreaticoduodenectomy; O-PPPD, open pylorus-preserving pancreaticoduodenectomy.

^a^ Others: The list of conditions including metastatic, GIST, and mucinous cystic neoplasm is not exhaustive.

**Table 2:** Final pathologic diagnosis of the lesions in the minimally invasive pylorus-preserving pancreaticoduodenectomy cohort.

Final pathology	Number (*n* = 391^a^)
Intraductal pancreatic mucinous neoplasm	102 (26.1%)
Pancreatic ductal adenocarcinoma	50 (12.8%)
Cholangiocarcinoma	31 (7.9%)
Solid pseudopapillary neoplasm	32 (8.2%)
Cystic neoplasm, cystadenoma, or serous cyst neoplasm	27 (6.9%)
Neuroendocrine tumor	31 (7.9%)
Pancreatic neuroendocrine tumor	16 (4.1%)
Ampullary carcinoma	13 (3.3%)
Ampulla of Vater carcinoma	25 (6.4%)
Periampullary pancreatic adenocarcinoma	7 (1.8%)
Chronic pancreatitis	6 (1.5%)
Duodenal gastrointestinal stromal tumor	5 (1.3%)
Mucinous cystadenoma	2 (0.5%)
Others	44 (11.3%)

^a^ Wang *et al* and Cai *et al* did not report the final MI-PPPD numbers therefore, the numbers from their studies were not accounted for.

**Table 3:** Comparison of surgical outcomes in the minimally invasive pylorus-preserving pancreaticoduodenectomy and open-pylorus-preserving pancreaticoduodenectomy cohorts. Data are presented in mean ± SD and *n*%.

Study ID	Primary outcome	Perioperative outcomes			Postoperative outcomes			
	*R*_0_ resection (*n*%)	Estimated blood loss (mL)	Operation time (min)	Length of hospital stay (days)	Pancreatic fistula (*n* %)	Delayed gastric emptying (*n* %)	Postpancreatectomy hemorrhage (*n* %)	90-day mortality, (*n* %)
Keck *et al* 2011^[24]^	MIS: 9, 100.0%	NR	MIS: 436.00 ± 28.00	NR	MIS: 2, 22.2%	MIS: 1, 11.1%	MIS: 1, 11.1%	MIS: 0
								O: 11, 3.2%
					O: 61, 17.0%	O: 35, 9.8%	O: 33, 9.2%	
	O: NR							
			O: 451.00 ± 109.00					
Wellner *et al* 2014^[22]^	MIS: 38, 95.0%	NR	MIS: 355.00 ± 229.10	NR	MIS: 7, 18.0%	MIS: 5, 15.0%	MIS: 5, 13.0%	MIS: 1, 3.0%
					O: 11, 28%	O: 11, 28.0%	O: 3, 8.0%	O: 0
	O: 38, 95.0%		O: 408.30 ± 365.20					
Morelli *et al* 2023^[23]^	NR	NR	MIS: 434 ± 60	MIS: 12.8 ± 9.5	MIS: 2, 6.7%	MIS: 3, 10%	NR	NR
					O: 2, 6.7%	O: 10, 33.3%		
			O: 409 ± 47	O: 16.5 ± 7.4				
Wan *et al* 2015^[20]^	NR	MIS: 269.17 ± 234.35	MIS: 506.67 ± 57.62	NR	MIS: 3, 30.0%	MIS: 0	NR	NR
						O: NR		
					O: NR			
			O: NR					
		O: NR						
Kim *et al* 2012^[25]^	MIS: 12, 12.0%	NR	MIS: 518.33 ± 406.19	MIS: 30.5 ± 49.65	MIS: 6, 6.0%	MIS: 2, 2.0%	MIS: 5, 5.0%	NR
							O: NR	
					O: NR	O: NR		
	O: NR		O: NR	O: NR				
Cho *et al* 2009^[26]^	NR	MIS: 445.00 ± 384.00	MIS: 338.00 ± 48.00	MIS: 16.4 ± 3.7	MIS: 2, 13.0%	MIS: 1, 7.0%	NR	NR
						O: 0		
		O: 552.00 ± 336.00						
					O: 2, 13.0%			
				O: 15.6 ± 1.3				
			O: 287 ± 117					
Song *et al* 2015^[27]^	MIS: 11, 100.0%	MIS: 609.00 ± 375.00	MIS: 482.50 ± 117.60	M: 14.3 ± 7.8	MIS: 6, 6.5%	MIS: 3, 3.2.0%	NR	NR
						O: 7, 7.5%		
	O: 218, 98.2%							
			O: 347.90 ± 97.20					
		O: 347.90 ± 87.20						
					O: 6, 6.5%			
				O: 19.2 ± 8.8				
Han *et al* 2019^[28]^	MIS: 100, 96.2%	MIS: 244.70 ± 189.30	MIS: 472.80 ± 70.80	MIS: 18.3 ± 13.9	MIS: 13, 13.5%	MIS: 16, 15.4%	MIS: 2, 1.9%	MIS: 1, 1.0%
							O: 2, 1.8%	
					O: 21, 18.8%			O: 0
						O: 19, 16.8%		
		O: 548.10 ± 419.30	O: 451.30 ± 105.50	O: 17.9 ± 7.7				
	O: 112, 99.1%							
Cai *et al* 2021^[21]^	NR	MIS: 396.67 ± 716.40	MIS: 300.4 ± 89.7	MIS: 11.33 ± 3.77	MIS: 3, 3.6%	MIS: 5, 6%	MIS: 0	MIS: 0
								O: NR
							O: NR	
					O: NR	O: NR		
			O: NR					
				O: NR				
		O: NR						

MIS, minimally invasive surgery; NR, no record; O, open; SD, standard deviation.

### Postoperative outcomes

Postoperative outcomes in this study included (1) the incidence of postoperative complications, with specific analyses for (2) the length of hospital stay, (3) pancreatic fistula formation, (4) DGE, (5) PPH, and (6) 90-day mortality. The following reported RR for postoperative complications were done based on the double-arm analysis between MI-PPPD and O-PPPD (Supplemental Digital Content Figure S3, available at: http://links.lww.com/JS9/G593). Four studies assessed the incidence of significant postoperative complications^[22,23,25,28]^ and found no difference between MI-PPPD and O-PPPD (RR = 1.00, 95% CI: 0.69; 1.46). Six studies reported the length of hospital stay^[22–26,28]^. Meta-analysis indicated no significant difference in the duration of hospital stay (MD = −0.21, 95% CI: −0.53; 0.11). Six studies reported the occurrence of pancreatic fistula formation^[22–26,28]^, and meta-analysis indicated no significant difference in the risk of POPF (RR = 0.78, 95% CI: 0.59; 1.05). The same six studies also examined DGE, and meta-analysis also indicated no significant difference in the risk of DGE (RR = 0.63, 95% CI: 0.35; 1.14). Three studies evaluated PPH, with meta-analysis exhibiting a lack of significant difference in the risk of haemorrhage (RR = 1.38, 95% CI: 0.77; 2.46)^[22,24,28]^. Similarly, three studies assessed 90-day mortality^[22,24,25]^, and no difference was observed in the meta-analysis of the risk of 90-day mortality (RR = 2.42, 95% CI: 0.92; 6.34).

### Perioperative outcomes

Perioperative outcomes of this study included (1) estimated blood loss, (2) operative time, and (3) conversion rate. The following MDs were based on the double arm analysis between MI-PPPD and O-PPPD (Supplemental Digital Content Figure S4, available at: http://links.lww.com/JS9/G593). Three studies evaluated estimated blood loss^[25,26,28]^. Meta-analysis indicated no statistically significant reduction in estimated blood loss in the MI-PPPD group (MD = −129.07, 95% CI: −579.35; 321.20). Operative time was reported by all six double-arm studies^[22–26,28]^. Meta-analysis reveals comparable surgical time across both groups (MD = 0.38, 95% CI: −0.18; 0.95). Five studies reported the conversion rate from MI-PPPD to O-PPPD^[20–24]^. Systematic review of these papers depicted a range of conversion rates from 0.0 to 40.0%, with a good spread of results at 1.2, 10.0, and 33.3%.

### Oncological outcomes

Oncological outcomes include (1) *R*_0_ resection rate, which indicates the proportion of patients with negative microscopic surgical margins, and (2) lymph nodes dissected yield, an objective measure of resection adequacy (Supplemental Digital Content Figure S5, available at: http://links.lww.com/JS9/G593). Three studies were included to evaluate the *R*_0_ resection rate between patients undergoing MI-PPPD and O-PPPD^[22,25,28]^. Meta-analysis of the three studies shows no significant difference in resection rate across both groups (OR = 0.50, 95% CI: 0.07; 3.82). Four studies were included to evaluate the number of lymph nodes harvested between patients undergoing MI-PPPD and O-PPPD^[22,23,25,26]^. Meta-analysis of the four studies also denotes a comparable number of lymph node yield across both groups (MD = −1.31, 95% CI: −2.83; 0.22) (Supplemental Digital Content Figure S5, available at: http://links.lww.com/JS9/G593).

## Discussion

Concerns about the safety and efficacy of MI-PPPD are material because the MIS approach intensifies the difficulty of the already highly complex PPPD, and the lack of evidence on the efficacy of MI-PPPD has limited the widespread adoption of this approach. PPPD requires preservation of the blood supply to the D1/2 segment of the small bowel to facilitate safe anastomosis via duodenojejunostomy. This requires preservation of the right gastroepiploic artery and right gastric artery to maintain blood supply to the duodenal stump, which will be used for duodenojejunostomy anastomosis. In addition, the ability to preserve the pylorus and D1 region relies heavily on whether the tumors in the head of pancreas region has invaded the antral-pyloric region of the stomach, as oncological clearance is of utmost importance and pylorus preservation is a secondary consideration. The intricacy and experience required to dissect around these structures and prevent anastomotic leaks make PPPD highly challenging. These challenges are further magnified in the MIS setting, especially in less experienced hands. The mirroring movement of the equipment in relation to the abdomen is not intuitive, and there is considerable discrepancy between the three-dimensional surgical space and the two-dimensional video footage projected by the laparoscope[29].

In light of increasing acceptance of PPPD as a viable option for pancreatic head resection due to its potential benefits in perioperative outcomes (i.e., reduced operative time and intra-operative blood loss), postoperative outcomes (i.e., decreased incidence of mortality, morbidity, postoperative pancreatic fistula, and improved postoperative weight gain), and comparable oncological outcomes^[7,11,30,31]^. This SRMA seeks to further evaluate these outcomes with the MIS approach for this surgery compared to the conventional open approach. Even though there are enormous technical challenges posed by MI-PPPD, the results indicate that there are no statistically significant differences in both perioperative and postoperative outcomes between MI-PPPD and O-PPPD, and therefore suggest noninferiority of MI-PPPD. One of the greatest strengths of MIS is that it provides a magnified view of the operating field, which may improve surgical precision and reduce unnecessary bowel manipulation[32]. This translates into reduced rates of bowel microtrauma, neighboring organ and neurovascular injury, reduced blood loss, and postoperative edema.

The results from this analysis seem to support the advantages that MIS confers on MI-PPPD. Nonstatistically significant improvement in estimated blood loss in MI-PPPD is not unexpected given the aforementioned advantages MIS confers, coupled with the tamponade effect from pneumoperitoneum used in MIS. Insignificant improvements in DGE may stem from more precise organ dissection that reduces vagal nerve injury[33] and postoperative peri-gastric edema. Less bowel injury and smaller incisions in MIS also lead to less postoperative pain[34] and reduce the need for pain-relieving opioids that suppress gastrointestinal motility and contribute to DGE. Intra-operative blood loss is an independent risk factor for POPF[35], and reduced blood loss in MIS ameliorates the rate of POPF. Improved anastomosis secondary to a better view of the bowel may help, too. DGE and POPF are two of the most important outcomes because DGE is a common criticism associated with PPPD, whilst improvement in POPF is expected in PPPD compared to PD. While insignificant and inconclusive, the underlying results hint at the potential MIS possesses in reducing the rates of DGE and extending the advantage of reduced rates of POPF in PPPD. Interestingly, the difference in operative time was insignificant as well, which refutes the notion that MIS is always slower than open surgery. This is consequential because operative time is a risk factor for surgical site infection, venous thromboembolism[36], and anesthesia-related complications, including hypotension, arrhythmias, atelectasis, and postoperative nausea and vomiting[37]. Possibilities for the indifference include significant time saved from quick access and closure of smaller incisions in MI-PPPD, execution of MI-PPPD by experienced surgeons in high-volume centers that could also reflect growing experience and confidence in the MIS approach, or inherent selection bias of nonadvanced pancreatic tumors in the MI-PPPD group.

In terms of oncological outcomes such as *R*_0_ resection rates and lymph node yield, there was also no significant difference between MI-PPPD and O-PPPD. Notably, most included studies reported a mean lymph node yield of 15 or above, meeting the established cut-off for oncological surgical adequacy for pancreatic cancers[38]. One study did not achieve a yield of at least 15 lymph nodes, but reported inadequate lymph node harvest across both the MI-PPPD group and O-PPPD group. Thus MI-PPPD appears to be noninferior in oncological outcomes. Establishing noninferior oncologic outcomes for MI-PPPD is essential, as the equivalence between PPPD and PD has previously justified the use of PPPD in pancreatic cancer resections. However, it must also be noted that MI-PPPD is often done for less complex cases where the tumors have not invaded the antral-pyloric region, which often denotes smaller tumors. The included studies reflect this, as there were few advanced pancreatic tumors in the cohort.

This paper posits that the nonsuperiority of MI-PPPD lies in its enormous technical demands and an overall lack of surgical proficiency to meet them. The current lack in surgical proficiency in MI-PPPD is supported by current literature that laparoscopic head of pancreas resections (which includes PPPD) have not overtaken their open counterparts, unlike distal pancreatectomies, due to the greater technical requirements of dissecting around neighboring major vessels^[39,40]^. The steep learning curve in minimally invasive pancreaticoduodenectomy (MI-PD) is often associated with suboptimal outcomes during the early phases of adoption, which require a substantial case volume to train the surgeons and achieve proficiency[41]. Scrutiny of the publications included in this meta-analysis reveal that these centers have not performed a high volume of such cases, with the majority of them below 40 cases (only one center performed 104 cases, and three centers between 80 and 100 cases). This might have contributed to a lack of statistically significant improvement in surgical outcomes in this meta-analysis and indicates a correlation between experience and efficacy of a surgical procedure.

Nonetheless, the efficacy of MI-PPPD may possibly supersede O-PPPD with sufficient time and training. Many MIS procedures have undergone similar milestones before becoming the standard of care in surgical practice. One such example is MI-PD that preceded PPPD. The early phase of MI-PD was concordant with that of MI-PPPD, where literature denoted poorer outcomes compared to the open approach, but the more recent studies tend to favor the MIS approach. Older systematic reviews found no significant differences between the MIS and open techniques in PD surgeries – the first traceable and conclusive SRMA of PD in March 2017 concluded that outcomes were, at best, comparable in high-volume centers but worse in low-volume centers[42]. This also suggests that experience and mastery are substantial drivers of improved efficacy in the MIS approach. This is further reinforced by Van Hilst’s robust multicenter patient-blinded randomized controlled study that highlighted increased mortality of laparoscopic PD performed by less experienced surgeons[42]. Kim *et al*^[43]^ depict the progressive nature of MIS with its findings that laparoscopic PD failure rates plateau after 42 cases and stabilize after 70 cases^[12,43,44]^. This also relates to the aforementioned low case volume and surgical inexperience in the context of MI-PPPD that plagued its performance. That said, the noninferior results from this study may suggest that MI-PPPD has progressed past the initial phase of poorer results and is currently in the phase of improving performance with an upwards trajectory. Therefore there is a reasonable basis to believe that MI-PPPD can surpass O-PPPD.

While surgeons are naturally concerned with surgical and oncological efficacy, the choice of surgical approach must be patient-centric. A holistic approach that considers the factors that matter to patients, especially cost, should be adopted[45]. Both approaches should be presented clearly to patients as options with comparable outcomes. The difference is that O-PPPD is more cost-friendly, whilst MI-PPPD potentially provides better quality of life outcomes. Surgeon’s preference and confidence may also be considered as a lack of confidence, which results in indecision and severe risk-averse behavior, which are detrimental to good surgical outcomes[46]. Surgeons may feel more comfortable and confident with the open approach, given the directness it offers and the relative ease of the approach. Given the extensive potential benefits that both PPPD and the MIS approach may confer, and the potential upwards trajectory of MI-PPPD to exceed O-PPPD, considerations to direct resources toward refining surgical techniques and enhancing surgeon training in MI-PPPD are recommended. Strategies to enhance surgeon training and mastery in MIS pancreatic surgery may follow a three-stage approach – skills acquisition, supervised practice, and then independent practice. For skills acquisition, simulator-training and attending MI-PPPD masterclasses to reference the subtleties applied by more experienced surgeons to improve operative outcomes may be done[47]. In the supervised practice phase, surgeons may perform MI-PPPD with an experienced surgeon in attendance to guide the training surgeon in real-time and increase the learning yield of each training surgery[46]. At an institutional level, the department may consider establishing a training framework that incorporates these strategies and ensures progressive clinical improvement for each of its surgeons.

A major strength of this study lies in its novelty – this is, to our knowledge, the first systematic review and meta-analysis that directly compares outcomes between MI-PPPD and O-PPPD. Whilst previous meta-analyses on PD have included PPPD data as a subset, none have directly and exclusively evaluated MI-PPPD against O-PPPD as this study does. Pooling together data across multiple databases and incorporating different study designs enhances the statistical power and breadth of the analysis of PPPD outcomes between the different approaches. Additionally, a wide range of clinically relevant outcomes was assessed, which provides a comprehensive overview of the comparative effectiveness and safety profile of MI-PPPD and O-PPPD.

However, this study is not without limitations. First, high heterogeneity was observed in the reporting of outcomes, which could not be fully accounted for. This is likely due to the variation of patient demographics and disease factors across studies. Differences in institutional practices, regional protocols, regional surgical expertise, and, in particular, the steep learning curve could have further contributed to the aforementioned heterogeneity. This gives rise to reduced confidence in the pooled estimates and a wider confidence interval, which could have contributed to the statistically insignificant results. This may be overcome by performing sensitivity analyses to remove outlier studies or performing subgroup analysis of studies with more homogenous outcomes. However, subgroup analysis was not feasible. Second, there were no randomized controlled trials (RCTs) analyzed in this paper. Instead, this paper analyzed observational studies, which inherently possess a higher risk of bias compared to RCTs. Unfortunately, there is no way to mitigate this limitation given the lack of RCTs conducted amidst the current available literature. Third, the sample size of patients undergoing MI-PPPD is slightly smaller than the sample size of patients undergoing O-PPPD in our analyses. This is likely due to the relatively recent adoption of fully minimally invasive techniques in PPPD, which could have led to an underrepresentation of the group of patients undergoing MI-PPPD. Fourth, while “quality of life” benefits were discussed in the paper, a proper analysis of such outcomes was not performed due to the limited available data. Furthermore, there were few advanced pancreatic tumors in the included studies. The relevance of any conclusions made on oncological outcomes assessment is limited to less advanced pancreatic tumors. Finally, the assessment of oncological outcomes was not comprehensive. The included studies lacked details on the type of surgical margin evaluated by pathologists. Given the limited current literature, there is a clear need for high-quality randomized controlled trials comparing MI-PPPD and O-PPPD. The conclusive role of MI-PPPD in replacing O-PPPD should be supported through these appropriate high-strength prospective randomized controlled studies that directly compare both procedures.

## Conclusion

Whilst MI-PPPD has not surpassed its open counterpart like many other procedures have, it is noninferior and thus, is likely to be in the improvement phase, whereby potential for superiority remains. However, such conclusions naturally hinge on future randomized, standardised, and multicentred trials. Until MIS is the clear “gold standard” for PPPD, the role of O-PPPD remains. Due consideration for channeling resources into MI-PPPD training is recommended, given its potential and the strong evidence correlating experience to improved outcomes for minimally invasive procedures.

## Data Availability

All data are publicly available.

## Research registration unique identifying number (UIN)

Registered on PROSPERO (reference: CRD420251013348, registered 2 April 2025).
